# Offspring pay sooner, parents pay later: experimental manipulation of body mass reveals trade-offs between immune function, reproduction and survival

**DOI:** 10.1186/1742-9994-10-77

**Published:** 2013-12-17

**Authors:** Arne Hegemann, Kevin D Matson, Heiner Flinks, B Irene Tieleman

**Affiliations:** 1 Animal Ecology Group, Centre for Ecological and Evolutionary Studies, University of Groningen, P.O. Box 11103, 9700, CC Groningen, The Netherlands; 2 Am Kuhm 19, 46325, Borken, Germany

**Keywords:** Birds, Cost of reproduction, Ecoimmunology, Ecophysiology, Immunity, Life history, Carry-over effect, Avian

## Abstract

**Introduction:**

Life-history theory predicts that organisms trade off survival against reproduction. However, the time scales on which various consequences become evident and the physiology mediating the cost of reproduction remain poorly understood. Yet, explaining not only which mechanisms mediate this trade-off, but also how fast or slow the mechanisms act, is crucial for an improved understanding of life-history evolution. We investigated three time scales on which an experimental increase in body mass could affect this trade-off: within broods, within season and between years. We handicapped adult skylarks (*Alauda arvensis)* by attaching extra weight during first broods to both adults of a pair. We measured body mass, immune function and return rates in these birds. We also measured nest success, feeding rates, diet composition, nestling size, nestling immune function and recruitment rates.

**Results:**

When nestlings of first broods fledged, parent body condition had not changed, but experimental birds experienced higher nest failure. Depending on the year, immune parameters of nestlings from experimental parents were either higher or lower than of control nestlings. Later, when parents were feeding their second brood, the balance between self-maintenance and nest success had shifted. Control and experimental adults differed in immune function, while mass and immune function of their nestlings did not differ. Although weights were removed after breeding, immune measurements during the second brood had the capacity to predict return rates to the next breeding season. Among birds that returned the next year, body condition and reproductive performance a year after the experiment did not differ between treatment groups.

**Conclusions:**

We conclude that the balance between current reproduction and survival shifts from affecting nestlings to affecting parents as the reproductive season progresses. Furthermore, immune function is apparently one physiological mechanism involved in this trade-off. By unravelling a physiological mechanism underlying the trade-offs between current and future reproduction and by demonstrating the different time scales on which it acts, our study represents an important step in understanding a central theory of life-history evolution.

## Introduction

The trade-off between current and future reproduction is central in life-history theory [1,2] and has been documented for many taxa including insects, fishes, reptiles, birds and mammals [3-5]. This trade-off can have consequences on different time scales, quantified mainly in studies on birds. For example, manipulating this trade-off via reproductive effort can directly affect nestlings and lead to reduced mass gain or increased mortality [6,7]. However, effects on the manipulated adults might develop more slowly and may become visible only after the breeding season [8]. Increased adult mortality often occurs in the subsequent winter [9-13]. Several physiology systems have been suggested to mediate the cost of current reproduction, especially the immune system may be an important mechanism [14,15], but unequivocal evidence is still lacking. Understanding not only which mechanisms mediate trade-offs, but also how fast or slow the mechanisms act, is crucial for an improved understanding of life-history evolution.

Despite the evidence that consequences of a shift in the trade-off between reproduction and self-maintenance can occur on different time scales, apparently no single study has investigated the underlying physiological mechanisms at multiple time levels. Likewise, no experimental study of the trade-off between reproduction and self-maintenance has linked changes in immune function to subsequent survival probabilities in both adults and their offspring. Many studies on the trade-off between reproduction and self-maintenance focus only on one time point: current reproduction [16-19]. A few studies include parameters from a second time point, which are typically reproductive parameters of subsequent reproductive attempts [20-22] or adult condition and performance parameters in the following year [23,24]. Changes in parental effort that affect future survival probabilities [9,23] may be mediated by changes in immune function. Trade-offs between reproduction and immune function are well established [15,24-28], and increased parasite infection rates in birds raising enlarged broods have also been described [14,15,28].

Studying the costs of reproduction and the underlying mechanisms requires an experimental approach. One way to influence the costs of reproduction involves manipulating the costs of locomotion (e.g. walking and flying) [16,18]. For example, handicapping birds with extra weight leads to increased locomotion costs [29-31]. Manipulating costs of locomotion might also affect investment in other physiological systems, such as the immune system, which has its own energetic demands [32]. Modulations of immune function by birds during periods of high locomotory costs [33,34] and of intense locomotory activity [34-37] are well established. Hence, manipulating locomotion costs of breeding birds provides the opportunity to study the balance between reproductive investment and self-maintenance with a consideration of possible immunological mechanisms.

We present a comprehensive immunological and behavioural dataset on skylarks (*Alauda arvensis)* with the aim of understanding trade-offs between parental investment in reproduction and self-maintenance along a time axis. We manipulated movement costs in free-living birds by handicapping them with extra weight, and we measured a variety of fitness-related parameters over three different time scales: a) the short-term effects within a breeding attempt, b) the medium-term effects on second broods within the same season and c) after removing the extra weight, the carry-over effects on return rates, immune function and reproduction in the subsequent year. We measured multiple immunological indices in the parents to quantify investment into self-maintenance at each of these time points and to correlate these with future return rates. We quantified current reproduction by measuring number and size of offspring. To explore whether nestlings differed beyond size and fledging rate, we also quantified parameters related to nutrition (feeding rates, diet composition), immune function and recruitment. We expected handicapped adults either to reduce investment in immune function, which might impair survival, or to reduce investment in reproduction, which might hinder nestling quality and recruitment. Within control birds, we did not expect a shift in parental investment from first to second broods, because in skylarks there is no clear trend for early- or late-born nestlings having different fitness benefits (Hegemann et al. unpublished data).

## Results

### Within-brood effects

#### Adult level

**Figure 1:**
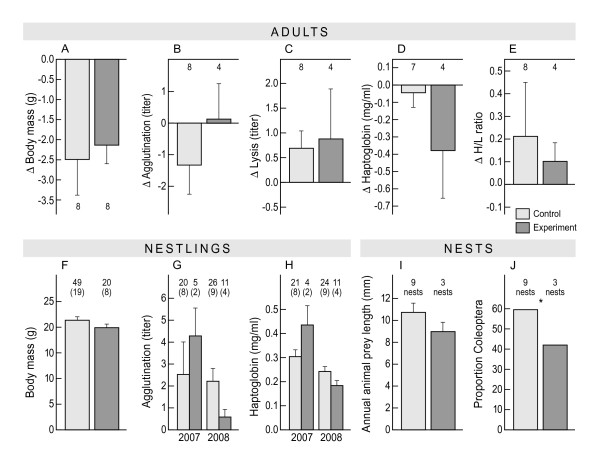
**Short-term (within-brood) effects of an experimental handicap on the trade-off between reproduction and self-maintenance in skylarks. A) – E)** Adult body mass and immune parameters. Values are expressed as the difference between the baseline measure taken when their nestlings were small, and the final measure taken when their nestlings were about to fledge. **F) – H)** Nestling body mass and immune measures from control and experimental parents; the latter were assigned to treatment groups 0-7 days earlier. **I)** Average length of animal prey in droppings of nestling skylarks. **J)** Proportion of the main prey type (beetles, order Coleoptera) in the diet of nestlings. Bars depict mean and standard error. Numbers represent sample size of individual birds. For nestlings the number of nests is given in parentheses. Stars denote statistically significant differences. If both years are plotted the interaction between year and treatment was significant. Statistical analyses can be found in Results and Additional file 1: Table S1.

The short-term handicap did not lead to significant differences between treatment groups of adults with respect to body mass, lysis titres, agglutination titres, haptoglobin concentrations, proportions of heterophils, lymphocytes, eosinophils, monocytes and the H/L-ratio when measured, on average, 6.5 (range 5-9) days after initiation of the experiment (always *P* > 0.18, *F* < 2.06; Figure 1A-F; Additional file 1: Table S1).

#### Nest level

The short-term handicap had moderate effects on nest success measures. Control nests had a success rate of 76% (19 out of 25) compared with 47% (8 out of 17) for experimental nests, but this difference was borderline non-significant (*χ*^*2*^ = 3.69, *P* = 0.055). As a consequence control pairs produced more fledglings (2.0 fledglings/control nest, 1.2 fledglings/experimental nest; *χ*^*2*^ = 4.14, n = 39, *P* = 0.042). Restricting the comparison to successful nests only, we found no difference in fledgling numbers between treatment groups; both produced on average 2.6 fledglings (n = 19 control nests, 8 experimental nests; *χ*^*2*^ = 0.02, *P* = 0.89). Control nests produced 0.22 recruits per fledgling (n = 13 recruits) and experimental nests 0.11 (n = 3 recruits) (*χ*^*2*^ = 0.82, *P* = 0.37). Feeding rates equalled 9.9 ± 1.34 visits per hour in the control (n = 11 nests) and 11.8 ± 2.20 visits per hour in the experimental group (n = 9 nests; *χ*^*2*^ = 0.50, *P* = 0.48). We found no significant differences between treatment groups in size (*F*_1,13_ = 0.51, *P* = 0.49, Figure 1I), number (*χ*^*2*^ = 0.43, *P* = 0.57) or diversity (*χ*^*2*^ = 0.25, *P* = 0.61) of prey items fed to nestlings, but the proportion of the main prey item (beetles, order Coleoptera) was significantly lower in the diet of experimental nestlings than in the diet of control nestlings (*χ*^*2*^-test, *χ*^*2*^ = 6.9, *P* = 0.008, Figure 1J).

#### Nestling level

The short-term handicap impacted nestling quality. Nestlings raised by experimental parents had higher agglutination titres and higher haptoglobin concentrations than those raised by control parents in 2007, but this pattern was reversed in 2008 (year*treatment interaction *χ*^*2*^ = 4.84, *P* = 0.028, Figure 1G and *χ*^*2*^_1_ = 4.05, *P* = 0.044; n = 62 nestlings, Figure 1H). Nestlings of experimental parents were 7.9% lighter than control nestlings (Figure 1F), but this difference – consistent in 2007 and 2008 – was not significant (*χ*^*2*^ = 1.66, n = 69, *P* = 0.19).

### Within-season effects

#### Adult level

**Figure 2:**
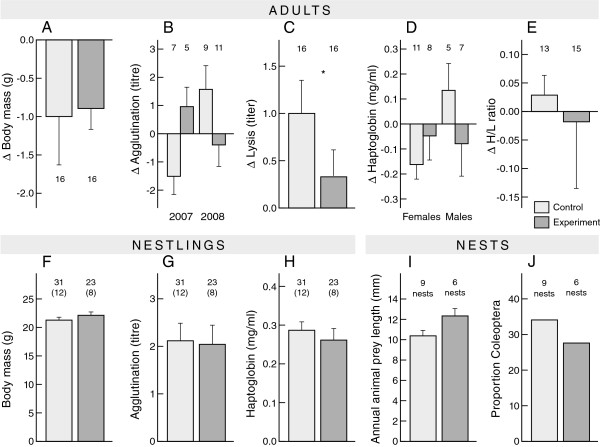
**Medium-term (within-season) effects of an experimental handicap on the trade-off between reproduction and self-maintenance in skylarks. A) – E)** Adult body mass and immune parameters ca. 5 weeks days after experimental initiation. Values are expressed as the difference between second and first broods. **F) – H)** Nestling body mass and immune measures in the offspring from control and experimental parents; the latter were assigned to treatment during first broods. **I)** Average length of animal prey in droppings of nestling skylarks. **J)** Proportion of the main prey type (beetles, order Coleoptera) in the diet of nestlings. Bars depict mean and standard error. Numbers represent sample size of individual birds. For nestlings the number of nests is given in parentheses. Stars denote statistically significant differences. If both years or sexes are plotted, then the interaction with treatment was significant. Statistical analyses can be found in Results and Additional file 1: Table S2.

In adult skylarks, the experimental treatment had a significant effect on agglutination and lysis titres, with the effect on agglutination being dependent on year (Figure 2B,C). Agglutination titres decreased in 2007 in control birds, but increased in experimental birds, while this pattern was reversed in 2008 (treatment*year interaction: *F*_1,26_ = 5.27, *P* = 0.030). Lysis titres increased in both groups from first to second broods but the increase was significantly weaker in experimental birds than in control birds (*F*_1,27_ = 4.79, *P* = 0.037). Haptoglobin concentrations were affected by treatment and sex (treatment*sex interaction: *F*_1,24_ = 5.85, *P* = 0.023; Figure 2D). In females, haptoglobin concentrations decreased more strongly in control birds than in experimental birds, while concentrations in control males increased and in experimental males decreased. In both groups the change in proportion of lymphocytes and eosinophils was negatively correlated with baseline values. However, this correlation was stronger in experimental birds (treatment*baseline *F*_1,23_ = 7.41, *P* = 0.012 for lymphocytes and *F*_1,23_ = 5.24, *P* = 0.031 for eosinophils). From first to second brood, adult skylarks exhibited decreased body mass (Figure 2A), increased proportions of heterophils and stable H/L-ratios (Figure 2E) and proportions of monocytes, but experimental and control birds did not differ in any of these changes (always *P* > 0.23, *F* < 1.53, Additional file 1: Table S2).

#### Nest level

The probability of nest success during the second broods differed between treatment groups depending on year. In 2007, 78% of control nests were successful compared with 25% of experimental nests. In 2008, 62% of control and 87% of experimental nests were successful (interaction year*treatment *χ*^2^ = 4.52, *P* = 0.033). Restricted to successful nests, number of fledglings did not differ between control (3.3 fledglings/successful nest, n = 12 nests) and experimental nests (3.4 fledglings/successful nest, n = 8 nests) (*χ*^*2*^ = 0.11, *P* = 0.74). The number of recruits per fledgling was 0.10 for control nests and 0.15 for experimental nests, a non-significant difference (*χ*^*2*^ = 0.41, n = 17, *P* = 0.52). The droppings of experimental nestlings contained remains of longer animal prey than control groups, a non-significant trend (*F*_1,13_ = 4.40, *P* = 0.056, Figure 2I). The number of animals (F_1,13_ = 1.81, *P* = 0.20), the diversity of prey (*F*_1,13_ = 2.28, *P* = 0.13) and the proportion of the main prey item (beetles, order Coleoptera) did not differ between groups (*χ*^*2*^-test, *χ*^*2*^ = 2.5, *P* = 0.12, Figure 2J).

#### Nestling level

Body mass (*χ*^*2*^ = 0.89, n = 52, *P* = 0.34), agglutination titre (*χ*^*2*^ = 0.60, n = 53 *P* = 0.44) and haptoglobin concentration (*χ*^*2*^ = 0.05, n = 53 *P* = 0.82) of nestlings did not differ between treatments (Figure 2F-H).

### Carry-over effects

In 2008 return rates of previously handicapped birds were considerably lower than of control birds (40.0% versus 85.7%, n = 2/5 versus 6/7), while in 2009 72.7% of experimental birds (n = 8/11) and 66.6% of control birds (n = 6/9) returned (interaction treatment*year *χ*^2^ = 2.22, *P* = 0.14). Combining both years, previously handicapped birds showed lower return rates than control birds (62.5% versus 75%) but the difference was not significant (*χ*^2^ = 0.97, *P* = 0.33). In the year following the experiment, returning birds did not differ between treatment groups in reproductive parameters, and recaptured birds did not differ between treatment groups in physiological parameters (Table 1). There was no relationship between the magnitude of change in any physiological parameter during the experiment and its value in the following year (always *P* > 0.27, *F* < 1.38, Additional file 1: Table S3), e.g. birds that lost more mass during the experiment were not necessarily the lightest ones in the following year.

**Table 1:** Carry-over effects on body mass, immune parameters and reproductive measures in the year following the experiment

**Parameter**	**Control mean ± se**	**Experimental mean ± se**	**N (Control/ Experimental)**	**F/Chisq**	**P**
Body mass (g)	32.5 ± 1.3	34.6 ± 1.1	11(5/6)	2.54	0.15
Lysis (titer)	0.45 ± 0.23	1.54 ± 0.43	11(5/6)	4.43	0.06
Agglutination (titer)	3.8 ± 0.2	4.5 ± 0.4	11(5/6)	1.77	0.22
Haptoglobin (mg/ml)	0.33 ± 0.04	0.39 ± 0.03	11(5/6)	0.65	0.44
H/L ratio	0.27 ± 0.06	0.61 ± 0.23	11(5/6)	0.98	0.36
Heterophils	17.2 ± 2.4	24.6 ± 5.7	11(5/6)	2.27	0.17
Lymphocytes	67 ± 5.6	50 ± 6.5	11(5/6)	1.28	0.30
Monocytes	4.6 ± 2.2	5.2 ± 0.7	11(5/6)	2.66	0.29
Eosinophils	11.2 ± 3.5	13.2 ± 3.2	11(5/6)	0.00	0.98
Nest success/attempt	27.8%	42.1%	37(18/19)	1.20	0.27
Fledglings/successful brood	3.4 ± 0.40	3.75 ± 0.16	13(5/8)*	0.72	0.40
Nestling body mass	23.1 ± 0.55	22.4 ± 0.52	44(17/27) [13(5/8)^1^]	0.85	0.36
Nestling agglutination (titer)	2.0 ± 0.45	1.6 ± 0.36	40(15/25) [13(5/8)^1^]	1.17	0.28
Nestling haptoglobin (mg/ml)	0.23 ± 0.01	0.24 ± 0.02	42(15/27) [13(5/8)^1^]	0.24	0.63

Shown are mean values, standard errors and sample sizes per treatment group. Statistical analyses can be found in Results and in Additional file 1: Table S3. *Number of successful nests, not number of fledglings; ^1^number of nests.

### Prediction of survival by immune function

**Figure 3:**
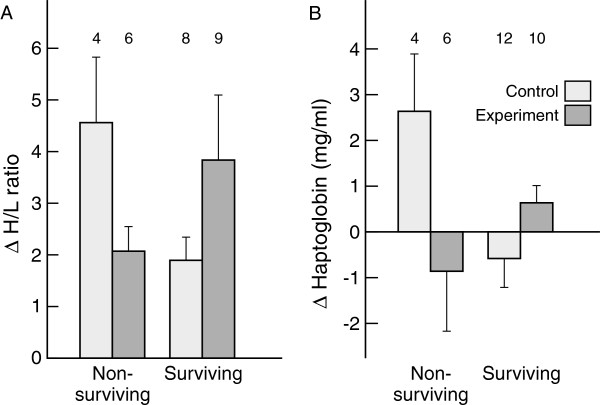
**Prediction of return rates by immune parameters. A)** Heterophil/lymphocyte-ratio at second brood and **B)** change in agglutination from first to second brood for experimental and control skylarks that returned the year after and for birds that did not return. The interaction between return rate and treatment was significant in both cases (P = 0.01). Statistical analyses can be found in Results.

We explored if the immune parameters of adult skylarks during rearing of the second brood (i.e., measured from samples collected at the point of removing extra weights from experimental birds) differed between birds that returned in the next year and birds that did not return, taking into account possible differences between treatment groups (treatment*immune interaction). Returning birds and non-returning birds differed in H/L-ratio and agglutination, but the direction of the effect depended on treatment (Figure 3). Returning birds and non-returning birds did not differ in any of the other immune parameters (always *χ*^*2*^ < 1.25, *P* > 0.26). Returning control skylarks had lower H/L-ratios at the end of the experiment than non-returning birds. This pattern was reversed in experimental birds (treatment*H/L-ratio: *χ*^*2*^ = 6.58, n = 23, *P* = 0.010). This interaction occurred with both the proportion of heterophils (*χ*^2^ = 6.01, *P* = 0.014) and lymphocytes (*χ*^2^ = 4.33, *P* = 0.037). A similar trend occurred in agglutination titres at the end of the experiment (treatment*agglutination: *χ*^2^ = 3.67, n = 27, *P* = 0.055). The change in agglutination titre during the experiment from first to second brood predicted return rates in control birds differently than in experimental birds (treatment*delta agglutination titre: *χ*^2^ = 6.55, *P* = 0.010; Figure 3B). Returning control birds decreased agglutination titres during the experiment, while non-returning birds increased agglutination titres; experimental birds showed the opposite pattern.

## Discussion

Skylarks handicapped by an extra weight modulated the trade-off between parental effort and investment into immune function differently at different time scales. During first broods adults maintained their condition and the costs were paid by the offspring. During the second brood, after birds carried their extra weight for several weeks, the costs were shifted to the adults, affecting their body condition and their return rates to the following breeding season. The costs on reproduction during first broods were expressed by fewer successful breeding attempts of experimental pairs. Furthermore, experimental nestlings showed altered immune parameters which coincided with a different diet they received. These nestlings also had lower recruitment rates, but the difference was not significant and sample sizes were small. During second broods, handicapped adults invested similar into reproduction than control birds. They brought a similar diet to their nestlings and likewise, the immune function of their nestlings did not differ from control nestlings. Instead, adults paid the costs, reflected in changes in their immune function. After removal of the handicap, adult return rate was 12.5% lower, but again this was not significant and sample sizes were modest. However, split up by treatment group, immune parameters measured when handicaps were removed from experimental birds predicted local survival. This suggests that reduced return rates and changes in immune function are linked. In the breeding season one year after the experiment, the returning birds no longer differed by treatment group in terms of immune parameters or reproductive performance.

After attachment of the extra weights, experimental birds faced higher nest failure rates, their fledglings expressed altered immune responses and these fledglings were (statistically insignificant) less likely to be detected as recruits. However, we found no effects on the immune system or body mass in adults over this short term. This indicates that during first broods, skylarks shift the costs of increased work load onto the nestlings. Such a pattern has been described for several species [6,7,38,39] but is generally associated with long-lived rather than short-lived species [2,7]. However, we cannot exclude the possibility that either restricted sample sizes or the short handicap period also contributed to the lack of effects on adults during within-broods measurements.

By their second brood, handicapped adult skylarks modulated several of their own immune indices, but their parental effort was not different from controls. This result suggests that the costs shifted back to the parents while parental effort and thus nestling condition was maintained. To our knowledge, our study is the first to document a shift in the trade-off between reproduction and self-maintenance from first to second reproductive attempt of a season and reflected by physiological changes. In adult skylarks lysis titres increase in the course of the breeding season [34], but handicapped birds were apparently not able to raise their complement activity as much as control birds. This suggests that birds reduced their investment in immune function after we experimentally increased their costs of reproduction. The effect of our experimental manipulation on haptoglobin concentration was sex-specific. Across our skylark population, haptoglobin remains constant over the breeding season [34]. Males are highly aggressive against neighbouring males. Carrying an extra weight is expected to decrease manoeuvrability [40], and consequently handicapped males might be less competitive and may suffer from more injuries than control males. Injuries usually cause an inflammation and haptoglobin levels in skylarks decrease following an inflammatory response [41]. This may explain why experimental males showed decreased haptoglobin concentrations after carrying an extra weight.

Our results show that the increased locomotory costs during reproduction and the lowered investment into immune function have carry-over effects that relate to return probabilities for both adults and their offspring. Based on modest sample sizes, we found only insignificant trends towards reduced local survival in adults and reduced local recruitment rates in their fledglings. But in adults, return rates could be predicted by immune parameters measured at the end of the breeding season. Trade-offs between reproduction and immune investment [25-28,42], and links between immune function and survival [43,44] are well documented. However, studies linking trade-offs between reproduction and immune function with subsequent survival were missing so far. We show that skylarks modulated immune parameters when costs of reproduction increased and these immune parameters relate to subsequent return rates. This also builds a case that we measured true survival rather than dispersal, especially given that skylarks anyhow show hardly any breeding dispersal [45,46]. Thus, our findings build on the results of Daan et al. [9], who demonstrated that kestrels (*Falco tinnuculus*) show increased mortality during winter rather than emigration after having raised experimentally enlarged broods. Survival in these kestrels was related to energy expenditure during breeding and this result led to the hypothesis that increased work load might cause a “physiological weakening” mediated by reductions in immune function [47]. While their study lacked a mechanistic link, we provide evidence that changes in immune defences may act as a mediator. One year after our manipulations, we did not find any immunological or reproductive effects of the experiment in the surviving birds, while such carry-over effects are known for other species [23].

Experimental manipulations of parental effort often have no effect on offspring body mass or structural size [16,19,48]. These negative results are typically interpreted as maintenance of current parental effort. In our study, feeding rates of nestlings and body mass of fledglings did not differ between treatment groups. Despite this, nestlings did differ in terms of immunological indices, which suggest adjustments in parental effort. One mediator may be diet, since the immune system and its development require energy and specific nutrients [32]. Indeed, we found that handicapped parents brought a modified diet to their nestlings. During first broods, when nestlings had altered immune function, the diet of experimental nestlings contained a significant lower proportion of beetles, their main food item. This suggests that these adults were less selective when collecting food. This change in adult behaviour coincides with a change in nestling immune function. In second broods the proportion of the main food type did not differ anymore and neither did nestling immune function. This strongly suggests that the species composition of nestling diet is an important factor shaping nestling immune function. Changes in foraging behaviour have been described previously for manipulated birds [17,49-52], but clear links to the physiology of the nestlings have remained elusive. We shed light on these links by showing that dietary differences correlate with immunological effects and lowered recruitment rates.

## Conclusions

We demonstrated that skylarks modulate the trade-off between current reproduction and survival differently over short-, medium- and long-time scales. Further we provided evidence that investment into the immune system is one physiological mechanism that mediates survival in adults and recruitment of their offspring. Our study represents an important step in understanding physiological mechanisms underlying the trade-offs between current and future reproduction, and thus adds to our understanding of life-history evolution.

## Methods

### Birds and experimental treatments

We studied skylarks in the Aekingerzand, the Netherlands (N 52°55′; E 6°18′) in 2007, 2008 and 2009 using a colour-ringed study population [34,45]. The skylark is a temperate zone passerine that breeds on the ground. Each pair starts 2-5 breeding attempts per year between the end of April and the end of July to compensate high nest predation rates [34,53,54]. The rate of breeding failure is high; consequently most pairs have only zero, one, or two successful broods per year (three successes are exceptionally rare, Hegemann et al. unpublished). It is only possible to reliable catch both parents of a pair when they are feeding nestlings.

To initiate the experiment, adults were caught when feeding nestlings (mean = 3 days old, range: 1-8 days old) during the first half of the breeding season (21-April-2007- 31-May-2007 and 04-May-2008 – 10-June-2008). We refer to the data collected at this initial capture as baseline values and to the breeding attempt as first broods. We cannot exclude that single pairs initiated an earlier breeding attempt that failed during the egg stage and before we found the nest. However, we are confident, that all pairs had no earlier nest containing nestlings because feeding behaviour is more obvious to detect. As pairs were assigned alternately to control and experimental groups, an earlier failed nesting attempt should not introduce any bias to our experiment. We attached an extra weight to experimental birds with a figure-eight harness made of elastic cotton thread [31,55] before release. Ranging from 3.0 to 3.9 g (total weight), the extra weight equalled 10% of an individual’s body mass. Experimental birds were handicapped by the combined effects of carrying the extra weight and wearing the harness; control birds remained without harness or extra weight.

We included experimental birds and their nests in this study only when both parents of a nest received a handicap to avoid the possibility that an unhandicapped partner compensated for a handicapped one [18,56]. For controls we included birds when we caught both parents of a nests (n = 8 nests) and also birds and their nests when only one parent was captured (n = 8 nests). We have no indication that capture, blood sampling and ringing (the only procedures imposed on control birds) had an effect on adult behaviour and nestling provisioning. Nestlings from nests with only one parent captured did not differ from nestlings where both parents were caught (nestling body mass: 21.7 ± 3.5 versus 21.1 ± 4.1 g, *P* = 0.66; agglutination: 2.5 ± 2.8 versus 2.2 ± 2.1 titres, *P* = 0.99; haptoglobin: 0.26 ± 0.11 versus 0.29 ± 0.14 mg/ml, *P* = 0.29). Thus, inclusion of controls for which only one parent was caught should not substantively bias our results, but this inclusion will increase the robustness of our conclusions through an increased sample size.

After initiation of the experiment, adult birds were part of up to three different data subsets to measure effects of the handicap on the trade-off between reproduction and self-maintenance over different time scales (within-brood, within-season, carry-over). The first data subset, which was used to evaluate the short-term effects of the handicap, included adults that we sampled before their nestlings were 3 days old and that we resampled when the offspring were 7-11 days old (“within-brood” measurements, n = 6 experimental, n = 8 controls). Nestlings leave the nest when 8 or 9 days old and will be fed by the parents until about 30 days old.

To evaluate the longer-term effects of the handicap, we recaptured and resampled 16 control and 16 experimental birds approximately 5 weeks (control birds: median = 39.5 days, range: 28-73 days; experimental birds: median = 35 days, range 27-52 days) after the first capture and when they were feeding nestlings of their second brood (“within-season” measurements). Upon that capture, we removed the extra weight of experimental birds. The cryptic behaviour of skylarks and their well-hidden nests mean that nests depredated at the egg stage may have been missed. However, we are confident that we found all successful nests of our focal birds, as feeding events are more obvious. We found a second nest for 66% of all birds. The chance to find a second nest did not differ between treatment groups (*χ*^*2*^ = 0.46, N = 43, *P* = 0.50) or years (*χ*^*2*^ = 0.05, N = 42, *P* = 0.83).

To evaluate carry-over effects of the handicap on survival to the breeding season following the treatment (2008 and 2009, respectively), we examined return rates of adults (to estimate survival) and young (to estimate recruitment) by ring reading. Both natal and breeding dispersal is very limited in skylarks [45]. To further evaluate the returning birds (for all of which we also had within-season measurements), we measured reproductive output (see below), and in those birds that were successfully recaptured (5 control, 6 experimental), we re-measured body mass and immune parameters. Two experimental and two control pairs stayed together from one breeding season to the next; all other birds had a new partner.

### Sample and data collection

Adults were sampled upon each capture, and nestlings were sampled around 8 days of age. Blood samples (~100-150 μl from adults, ~ 70-100 μl from nestlings) were collected into heparinised capillary tubes from the brachial vein. Adults were bled directly after capture (median: 5 min; range: 3-15 min) and before any impacts of handling stress on immune parameters are expected [57,58]. Blood smears for leukocyte enumeration (adults only) were made from a drop of fresh blood. The remaining blood was stored on ice until centrifuged in the lab (10 min, 7000 rpm). Plasma was frozen for future analyses. Structural measurements and body mass were recorded after blood collection, and birds were ringed with metal and colour rings. Adult birds were sexed biometrically, nestlings were sexed molecularly [59].

We measured three general categories of immune defence. We used plasma to quantify titres of complement-like lytic enzymes (lysis) and non-specific natural antibodies (agglutination) [34,60]. Blood of 8-day-old nestlings did not show lytic activity (Hegemann et al. unpublished). We used a functional assay to measure haptoglobin-like activity (hereafter haptoglobin in mg/ml) [34,61]. In skylarks, haptoglobin decreases following an immune challenge [41]. Leukocyte proportions (lymphocytes, heterophils, basophils, monocytes or eosinophils) based on the first 100 white blood cells (WBC) were determined from blood smears by one person (C. Gotteland), who was blind to year and treatment. Leukocyte proportions reflect both innate and acquired components and change in response to immunological stimulation [62]. Analyses of leukocyte profiles include the ratio of heterophils and lymphocytes (H/L ratio) which is related to different types of stressors, including immunological ones [63]. In most blood smears (61%) no basophils were detected, so we did not analyse this cell type. We took biological and methodological factors into consideration when choosing to focus mainly on measures of innate immunity: This sub-system is an important first line of defence [64], and this importance might translate into consistency over longer time scales, a point that coordinates with our main hypothesis. Additionally, while measures of innate immunity can vary over shorter scales (e.g. reflecting current “health status” or “physiological condition,” [65], the absence of immunological memory in vertebrate innate sub-systems allows for interpretation of repeated samples without confounding the magnitude of an index and the exposure to a particular disease [66,67].

Nest success rates (at least one fledged nestling vs. nest failure) and number of fledglings were recorded on day 8. After ringing nestlings leave the nest. We measured feeding rates on first broods in 2008 (n = 14 nests) by observing nests with binoculars for one hour in the morning. Feeding rates (number of feeding events/hour) were measured when nestlings were 4 days (n = 5 control nests, 4 experimental nests) and 6-7 days (n = 6 control nests, 5 experimental nests) old.

Skylark nestlings usually defecate during ringing. To analyse nestling diet, we collected these droppings per nest, preserved them with table salt and froze them until analyses. Droppings of 27 nests (first broods: 9 control, 3 experimental; second broods: 9 control, 6 experimental) were analysed [68] by H.F., who was blind to brood and treatment. We summarized the dropping analyses in three variables per nest: number of animal prey individuals, average length of animal prey, and number of different prey types. We also compared the proportion of the main food type, beetles (order Coleoptera), between treatment groups. Animal prey length (reflecting biomass) was estimated from prey remains using a reference collection and information from literature [68-71].

### Statistics

We analysed data using R version 2.14.0 [72]. A detailed description of statistical methods can be found in Additional file 2 in the supporting information. Here, we give a brief summary of all statistical tests. For within-brood and within-season measurements, we used linear models and the differences between the two measurements as the dependent variables. We preferred calculating the difference between time points over using a repeated design in a mixed model, because the latter treats both time points equal, while we are specifically interested in the change of each response variable during the experiment. We included the baseline values of the corresponding response variable as a covariate to account for potentially different starting points among individuals. Including nest as a random effect (to account for possible non-independence of pair members) did not significantly improve the fit of any starting model (always p > 0.54), thus we decided for the simpler and hence more powerful linear models without nest as random effect. Nest success rates, number of fledglings and number of recruits per fledgling were analysed on the nest level with generalized linear models. Body mass and immune parameters of nestlings were analysed with linear mixed models, and feeding rates were analysed with generalized linear mixed models with a Poisson error structure, all including nest as a random effect to account for non-independence of siblings. Return rates of adults were analysed with generalized linear models with binomial error structure. We tested if returning could be predicted by any measurement at the end of the experiment (removal of weights from experimental birds). We did this by sequentially including the interaction between treatment and each response variable.

We included, when applicable, the following variables in each model: treatment, year, sex, baseline value, age of nestlings, number of nestlings and length of experiment (number of days between measurements). We also included two-way-interactions involving treatment. We simplified the starting models using backward elimination based on likelihood-ratio tests and F-statistics (Chisq-statistics for generalized linear models with binomial or poisson error structure and for mixed models) and with *P* < 0.05 as the selection criterion (“drop1”-function of R) until reaching the final model with only significant terms. Assumptions of all models were checked on the residuals of the final model. We report interactions only when significant. Full statistics of all main effects can be found in Additional file 1: Tables S1-S3. Treatment groups did not differ by chance in brood size, body mass or any immune parameter at the initiation of the experiment (always *P* > 0.21).

The study was performed under license D4743A and DEC5219C of the Institutional Animal Care and Use Committee of the University of Groningen.

## Competing interests

The authors declare that they have no competing interests.

## Authors’ contributions

AH and BIT conceived and designed the experiment. AH performed the experiment. AH did the immunological assays with help of KDM. HF analysed the dropping content. AH did the statistical analyses with advice of KDM and BIT. AH, BIT, KDM and HF wrote the paper. All authors read and approved the final manuscript.

## Supplementary Material

Additional file 1: Tables S1-S3 Detailed statistics and coefficients. Click here for file

Additional file 2 Details of statistical methods. Click here for file
