# Supplementary material for: Offspring pay sooner, parents pay later: experimental manipulation of body mass reveals trade-offs between immune function, reproduction and survival
Source: Front Zool. 2013 Dec 17;10:77. doi: 10.1186/1742-9994-10-77 (PMC3878409; doi:10.1186/1742-9994-10-77)
Supplement: Additional file 1: Tables S1-S3 — Detailed statistics and coefficients. [file 1742-9994-10-77-S1.pdf]

Offspring pay sooner, parents pay later: Experimental manipulation of body mass reveals trade-offs between immune function, reproduction and survival

Arne Hegemann[1*], Kevin D. Matson[1], Heiner Flinks[2] & B. Irene Tieleman[1]

Frontiers in Zoology

Corresponding author: Arne Hegemann, Animal Ecology Group, Centre for Ecological and Evolutionary Studies, University of Groningen, P.O. Box 11103, 9700 CC Groningen, The Netherlands, a.hegemann@rug.nl

Appendix2 : Statistics and coefficients

Table S1: (next pages) Statistics and coefficients of the linear models for within-brood analyses during a handicap experiment in Skylarks. Results are from linear models after removing all non-significant interactions (*P* > 0.05). *P*-values < 0.05 are bold. Beta values are only given for significant factors. A) Adults, B) Nest, C) Nestlings. Nest data were analysed with generalized linear models and the appropriate error structure. For nestlings and feeding rates, nest identity was included as random effect. For further details see statistic section in methods. t = number of days since baseline measurement; Baseline=measurement of y at the initial capture.

**A) Adults**							
y-variable	Source of variation	*df*	*F*	*P*	mean	beta	reference
Δ Body mass	…..				-2.34		
	Treatment	1	0.45	0.521			
	Year	1	0.34	0.574			
	Sex	1	1.18	0.299			
	t	1	0.31	0.591			
	Baseline	1	2.03	0.182			
Δ Lysis titre	…..				0.75		
	Treatment	1	0.16	0.699			
	Year	1	4.69	0.062			
	Sex	1	2.04	0.196			
	t	1	11.77	**0.008**		-0.78	
	Baseline	1	16.83	**0.003**		-0.90	
Δ Agglutination titre	…..				-0.83		
	Treatment	1	0.46	0.523			
	Year	1	14.89	**0.004**		2.19	2008
	Sex	1	3.19	0.112			
	t	1	0.15	0.710			
	Baseline	1	64.75	**<0.001**		-0.95	
Δ Haptoglobin	…..				-0.17		
	Treatment	1	0.28	0.622			
	Year	1	0.11	0.750			
	Sex	1	1.36	0.278			
	t	1	0.08	0.781			
	Baseline	1	1.86	**<0.001**		-1.06	
Δ Heterophils	…..				8.73		
	Treatment	1	0.01	0.946			
	Year	1	0.43	0.528			
	Sex	1	0.01	0.938			
	t	1	0.44	0.523			
	Baseline	1	0.23	0.642			
Δ Lymphocytes	…..				2.11		
	Treatment	1	0.03	0.874			
	Year	1	0.39	0.405			
	Sex	1	0.01	0.932			
	t	1	1.25	0.292			
	Baseline	1	3.98	0.074			
Δ Monocytes	…..				-1.39		
	Treatment	1	2.06	0.175			
	Year	1	0.63	0.451			
	Sex	1	0.08	0.791			
	t	1	0.76	0.410			
	Baseline	1	35.27	**<0.001**		-0.97	
Δ Eosinophils	…..				-9.15		
	Treatment	1	0.01	0.921			
	Year	1	0.01	0.928			
	Sex	1	0.13	0.730			
	t	1	0.40	0.542			
	Baseline	1	93.26	**<0.001**		-0.76	
Δ H/L-ratio	…..				0.17		
	Treatment	1	0.14	0.723			
	Year	1	0.65	0.444			
	Sex	1	0.01	0.938			
	t	1	1.13	0.311			
	Baseline	1	0.94	0.356			

B) **Nests**							
y-variable	Source of variation	*df*	χ^2/F	*P*	mean	beta	reference
Nest success	…..				0.64		
	Treatment	1	3.69	0.055			
	Year	1	2.23	0.135			
Number fledglings	…..				1.69		
(successful nests only)	Treatment	1	0.02	0.889			
	Year	1	0.00	0.999			
	Age when exp. started	1	0.70	0.404			
Recruits per fledgling	…..				0.2		
(successful nests only)	Treatment	1	0.82	0.366			
	Year	1	0.29	0.588			
	Age when exp. started	1	0.19	0.662			
Feeding rate	…..				10.75		
	Treatment	1	0.497	0.481			
	Age of Nestlings	1	8.50	**0.004**		0.46	
	t	1	0.24	0.627			
Droppings	…..				10.3		
(length animals)	Treatment	1	0.51	0.492			
	Year	1	0.31	0.591			
	Number nestlings	1	8.46	**0.016**		-2.1	
Droppings (no animals)	…..				25.03		
	Treatment	1	0.43	0.527			
	Year	1	0.00	0.960			
	Number nestlings	1	0.46	0.512			
Droppings	…..				9.67		
(prey diversity)	Treatment	1	0.25	0.614			
	Year	1	1.87	0.172			
	Number nestlings	1	4.86	**0.027**		0.32	

C) **Nestlings**							
y-variable	Source of variation	*df*	χ^2/F	*P*	mean	beta	reference
Body mass	….				21.06		
	Treatment	1	1.66	0.198			
	Year	1	0.46	0.498			
	Sex	1	36.95	**<0.001**		3.25	Male
	Number nestlings	1	4.35	**0.037**		1.30	
	Age nestlings	1	11.22	**<0.001**		3.74	
	Age at experimental initiation	1	0.33	0.567			
Agglutination titre	….				2.22		
	Treatment	1	See text for interaction				
	Year	1	See text for interaction				
	Sex	1	0.24	0.627			
	Number nestlings	1	0.39	0.533			
	Age nestlings	1	0.86	0.354			
	Age at experimental initiation	1	0.75	0.385			
Haptoglobin	….				0.27		
	Treatment	1	See text for interaction				
	Year	1	See text for interaction				
	Sex	1	0.62	0.430			
	Number nestlings	1	3.16	0.076			
	Age nestlings	1	1.18	0.276			
	Age at experimental initiation	1	0.00	0.983			

Table S2: (next pages) Statistics and coefficients of the linear models for within-season (second brood) analyses during a handicap experiment in Skylarks. Results are from linear models after removing all non-significant interactions ($P > 0.05$). P-values < 0.05 are bold. A) Adults, B) Nest, C) Nestlings. Nest data were analysed with generalized linear models and the appropriate error structure. For nestlings nest identity was included as random effect. For further details see statistic section in methods. t = number of days since baseline measurement; Baseline=measurement of y at the initial capture.

**A) Adults**							
y-variable	Source of variation	*df*	*F*	*P*	mean	beta	reference
Δ Body mass	…..				-0.95		
	Treatment	1	0.00	0.995			
	Year	1	1.26	0.271			
	Sex	1	5.30	**0.029**		1.57	Male
	t	1	7.52	**0.011**		-0.07	
	Baseline	1	9.29	**0.005**		-0.38	
Δ Lysis titre	…..				0.66		
	Treatment	1	4.79	**0.037**		-0.82	handicapped
	Year	1	5.15	**0.031**		-1.05	2008
	Sex	1	2.19	0.152			
	t	1	3.49	**0.073**			
	Baseline	1	4.78	**0.038**		-0.39	
Δ Agglutination titre	…..				0.15		
	Treatment	1	See text for interaction				
	Year	1	See text for interaction				
	Sex	1	0.02	0.902			
	t	1	0.64	0.432			
	Baseline	1	18.28	<**0.001**		-0.71	
Δ Haptoglobin	…..				-0.07		
	Treatment	1	See text for interaction				
	Year	1	12.59	0.002		-0.23	2008
	Sex	1	See text for interaction				
	t	1	0.12	0.729			
	Baseline	1	43.49	<**0.001**		-0.29	
Δ Heterophils	…..				0.03		
	Treatment	1	0.01	0.933			
	Year	1	1.47	0.237			
	Sex	1	1.01	0.325			
	t	1	0.04	0.837			
	Baseline	1	8.84	**0.006**		-0.61	
Δ Lymphocytes	…..				0.004		
	Treatment	1	See text for interaction				
	Year	1	2.86	0.105			
	Sex	1	1.74	0.201			
	t	1	0.22	0.644			
	Baseline	1	See text for interaction				
Δ Monocytes	…..				-0.0003		
	Treatment	1	1.53	0.228			
	Year	1	0.58	0.453			
	Sex	1	0.16	0.696			
	t	1	1.09	0.307			
	Baseline	1	32.99	<**0.001**		-1.19	
Δ Eosinophils	…..				0.03		
	Treatment	1	See text for interaction				
	Year	1	1.32	0.264			
	Sex	1	0.69	0.416			
	t	1	0.27	0.612			
	Baseline	1	See text for interaction				
Δ H/L-ratio	…..				0.04		
	Treatment	1	0.05	0.823			
	Year	1	2.29	0.143			
	Sex	1	0.77	0.388			
	t	1	0.05	0.818			
	Baseline	1	20.10	<**0.001**		-0.74	

**B) Nests**							
y-variable	Source of variation	*df*	χ^2/F	*P*	mean	beta	reference
Nest success	…..				0.69		
	Treatment	1	See text for interaction				
	Year	1	See text for interaction				
Number fledglings (successful nests only)	…..				2.28		
	Treatment	1	0.11	0.735			
	Year	1	0.03	0.801			
	Age of Nestlings	1	0.58	0.446			
	Age when parents recaptured	1	0.12	0.728			
Recruits per fledgling (successful nests only)	…..				0.21		
	Treatment	1	0.41	0.524			
	Year	1	0.01	0.926			
	Age of Nestlings	1	0.36	0.550			
	Age when parents recaptured	1	1.81	0.178			
Droppings (length animals)	…..				11.3		
	Treatment	1	4.40	**0.056**		2.02	handicapped
	Year	1	2.39	0.148			
	Number nestlings	1	0.29	0.601			
Droppings (no animals)	…..				33.0		
	Treatment	1	1.81	0.201			
	Year	1	0.04	0.836			
	Number nestlings	1	0.62	0.448			
Droppings (prey diversity)	…..				11.1		
	Treatment	1	2.28	0.131			
	Year	1	0.17	0.680			
	Number nestlings	1	0.71	0.400			

**C) Nestlings**							
y-variable	Source of variation	**df**	χ^2	*P*	mean	beta	reference
Body mass	….				21.62		
	Treatment	1	0.89	0.344			
	Year	1	1.85	0.174			
	Sex	1	15.11	**0.001**		2.65	Male
	Number nestlings	1	0.24	0.617			
	Age nestlings	1	0.03	0.859			
Agglutination titre	….				2.08		
	Treatment	1	0.60	0.438			
	Year	1	3.78	0.052			
	Sex	1	10.63	**0.001**		1.74	Male
	Number nestlings	1	0.37	0.534			
	Age nestlings	1	0.04	0.848			
Haptoglobin	….				0.28		
	Treatment	1	0.05	0.821			
	Year	1	2.74	0.097			
	Sex	1	6.27	**0.012**		-0.09	Male
	Number nestlings	1	0.19	0.658			
	Age nestlings	1	5.14	**0.023**		0.08	

Table S3: (next pages) Statistics and coefficients of the linear models for carry-over effects after a handicap experiment in Skylarks. Results are from linear models after removing all non-significant interactions (*P* >0.05). *P*-values < 0.05 are bold. A) Adults, B) Nest, C) Nestlings. Nest data were analysed with generalized linear models and the appropriate error structure. For nestlings, nest identity was included as random effect. For further details see statistic section in methods. t = number of days since baseline measurement; Baseline=measurement of y at the initial capture.

**A) Adults**							
y-variable	Source of variation	*df*	*F*	*P*	mean	beta	reference
Body mass	…..				33.65		
	Treatment	1	2.54	0.150			
	Year	1	0.11	0.753			
	Sex	1	23.80	**<0.001**		5.28	Male
	Length experiment	1	0.74	0.417			
	Δ body mass	1	0.02	0.882			
Lysis titre	…..				1.05		
	Treatment	1	4.43	0.065			
	Year	1	0.11	0.753			
	Sex	1	1.60	0.241			
	Length experiment	1	0.02	0.889			
	Δ lysis titre	1	1.16	0.317			
Agglutination titre	…..				4.18		
	Treatment	1	1.77	0.216			
	Year	1	0.458	0.520			
	Sex	1	0.11	0.747			
	Length experiment	1	0.07	0.800			
	Δ agglutination titre	1	1.00	0.346			
Haptoglobin	…..				0.36		
	Treatment	1	0.658	0.444			
	Year	1	2.53	0.146			
	Sex	1	0.52	0.502			
	Length experiment	1	2.09	0.186			
	Δ haptoglobin	1	1.15	0.324			
Heterophils	…..				21.3		
	Treatment	1	2.27	0.166			
	Year	1	0.07	0.794			
	Sex	1	0.26	0.624			
	Length experiment	1	0.01	0.915			
	Δ heterophils	1	0.02	0.904			
Lymphocytes	…..				57.7		
	Treatment	1	1.27	0.296			
	Year	1	3.87	0.081			
	Sex	1	5.15	0.053			
	Length experiment	1	0.03	0.867			
	Δ lymphocytes	1	0.23	0.651			
Monocytes	…..				4.9		
	Treatment	1	2.66	0.147			
	Year	1	0.03	0.878			
	Sex	1	7.08	**0.026**		0.96	Male
	Length experiment	1	1.91	0.216			
	Δ monocytes	1	1.38	0.274			
Eosinophils	…..				12.3		
	Treatment	1	0.00	0.975			
	Year	1	9.21	**0.014**		1.32	2009
	Sex	1	3.96	0.082			
	Length experiment	1	0.01	0.910			
	Δ eosinophils	1	0.30	0.603			
H/L-ratio	…..				0.46		
	Treatment	1	0.98	0.359			
	Year	1	1.57	0.250			
	Sex	1	0.52	0.503			
	Length experiment	1	0.02	0.896			
	Δ H/L-ratio	1	1.28	0.292			

B) Nests

y-variable	Source of variation	*df*	χ^2	*P*	mean	beta	reference
Nest success	…..				0.35		
	Treatment	1	1.20	0.273			
	Year	1	2.93	0.087			
Number fledglings	…..				1.3		
(all nests)	Treatment	1	0.72	0.395			
	Year	1	1.93	0.165			

C) Nestlings

y-variable	Source of variation	*df*	χ^2	*P*	mean	beta	reference
Body mass	….				22.7		
	Treatment	1	0.85	0.358			
	Year	1	10.38	**0.001**		2.32	2009
	Sex	1	41.79	<**0.001**		3.22	Male
	Number nestlings	1	2.50	0.114		1.83	
	Age nestlings	1	7.97	**0.005**			
Agglutination titre	….				1.8		
	Treatment	1	1.17	0.280			
	Year	1	2.27	0.132			
	Sex	1	1.86	0.173			
	Number nestlings	1	2.18	0.139			
	Age nestlings	1	1.28	0.258			
Haptoglobin	….				0.24		
	Treatment	1	0.24	0.626			
	Year	1	1.80	0.180			
	Sex	1	0.01	0.924			
	Number nestlings	1	0.18	0.670			
	Age nestlings	1	0.753	0.386			
