# Supplementary material for: Offspring pay sooner, parents pay later: experimental manipulation of body mass reveals trade-offs between immune function, reproduction and survival
Source: Front Zool. 2013 Dec 17;10:77. doi: 10.1186/1742-9994-10-77 (PMC3878409; doi:10.1186/1742-9994-10-77)
Supplement: Additional file 2 — Details of statistical methods. [file 1742-9994-10-77-S2.pdf]

Offspring pay sooner, parents pay later: Experimental manipulation of body mass reveals trade-offs between immune function, reproduction and survival

Arne Hegemann[1*], Kevin D. Matson[1], Heiner Flinks[2] & B. Irene Tieleman[1]

**Frontiers in Zoology**

**Corresponding author: Arne Hegemann,** Animal Ecology Group, Centre for Ecological and Evolutionary Studies, University of Groningen, P.O. Box 11103, 9700 CC Groningen, The Netherlands, a.hegemann@rug.nl

## Appendix1 : Details of statistical methods

We analysed data using R version 2.14.0 (R Development Core Team 2011). For within-brood and within-season measurements we used linear models with the difference between the two time points for every dependent variable. We always included treatment group, time since baseline measurement, sex and year. We also included baseline values as a fixed effect into the models to account for different starting values (e.g. birds with a high body mass might be able to lose more mass than initially light birds). Including nest as a random effect (to account for possible non-independence of pair members) did not significantly improve the fit of any starting model (always $p > 0.54$), thus we decided for the simpler and hence more powerful linear models without nest as random effect. We preferred calculating the difference between time points and using a linear model that includes the baseline as covariate over

using a repeated design in a mixed models, because the latter treats both time points equal, while we are specifically interested in the change of each response variable during the experiment. The interaction between treatment and time since baseline measurement was included in within-brood analyses. Interactions between treatment and sex, year and time since baseline measurement were included in within-season analyses. Within-brood analyses included the interaction between treatment and time since initiation of the experiment. Julian day had no effect on baseline or within-season data and was therefore not included in analyses. Data collected on birds that we recaptured the following year were analysed with linear models using the measurement taken at recapture as response variable and treatment, sex, year, length of the experiment in days (from initiation until extra weights were removed from experimental birds), and the change in the explanatory variable during the experiment, as predictors. Because response variables were not calculated as a difference between two time points, but included as single time point measurements, we analyzed the proportion of each WBC type against the total WBC number minus the focal WBC type using a binomial approach.

Nest success, number of fledglings and number of recruits were analysed on the nest level with generalized linear models and the appropriate corresponding error structure (binomial, poisson, Gaussian or quasibinomial and quasipoisson when overdispersion occurred). Treatment, year, age of nestlings when the experiment started (or stopped, for second broods) and the two-way-interaction between treatment and year were always included. Feeding rates were tested with a generalized linear mixed model with poisson error distribution and including nest as random effect to avoid pseudoreplication. Models included treatment, age of nestlings, days since initiation of the experiment and the two-way-interactions with treatment. The variables from the dropping analyses were tested in generalized linear models with year, treatment and number of nestlings as fixed effects and a

quasipoisson (number of animal prey), poisson (diversity of prey items) or Gaussian (length of prey items) error structure.

Individual nestling data were analysed with linear mixed models including sex, treatment, year, age, number of nestlings and age of nestlings when parents were captured as well as the two-way interactions of treatment with year and number of nestlings. Nest identity was always included as random factor to account for non-independence of siblings.

Survival and recruitment data were analysed with generalized linear models with binomial error structure and treatment, sex, year, length of the experiment and the two-way interactions involving treatment as fixed explanatory variables. We tested if survival could be predicted by any measurement at the end of the experiment by sequentially including the interaction of treatment with each measured immune parameter and body mass as well as their change during the experiment. As covariates we included the length of the experiment (in days), sex and year.

We always started with the full model and simplified it using backward elimination based on likelihood-ratio test and F-Statistics (Chisq-Statistics for generalized linear models with binomial or poisson error structure) and with $P < 0.05$ as the selection criterion ("drop1"-function of R) until reaching the minimal adequate model. Assumptions of all models were checked on the residuals of the final model. We report interactions only when significant.
